# Prolyl isomerase Pin1 is highly expressed in Her2-positive breast cancer and regulates erbB2 protein stability

**DOI:** 10.1186/1476-4598-7-91

**Published:** 2008-12-15

**Authors:** Prudence B Lam, Laura N Burga, Bryan P Wu, Erin W Hofstatter, Kun Ping Lu, Gerburg M Wulf

**Affiliations:** 1 Cancer Biology Program, Division of Hematology/Oncology, Department of Medicine, Beth Israel Deaconess Medical Center, Harvard Medical School, 330 Brookline Avenue, NRB 1030c, Boston, MA 02215, USA; 2 Cambridge Health Alliance, 1439 Cambridge St, Cambridge, MA 02139, USA

## Abstract

Overexpression of HER-2/Neu occurs in about 25–30% of breast cancer patients and is indicative of poor prognosis. While Her2/Neu overexpression is primarily a result of erbB2 amplification, it has recently been recognized that erbB2 levels are also regulated on the protein level. However, factors that regulate Her2/Neu protein stability are less well understood. The prolyl isomerase Pin1 catalyzes the isomerization of specific pSer/Thr-Pro motifs that have been phosphorylated in response to mitogenic signaling. We have previously reported that Pin1-catalyzed post-phosphorylational modification of signal transduction modulates the oncogenic pathways downstream from c-neu. The goal of this study was to examine the expression of prolyl isomerase Pin1 in human Her2+ breast cancer, and to study if Pin1 affects the expression of Her2/Neu itself.

**Methods:**

Immunohistochemistry for Her2 and Pin1 were performed on two hundred twenty-three human breast cancers, with 59% of the specimen from primary cancers and 41% from metastatic sites. Pin1 inhibition was achieved using siRNA in Her2+ breast cancer cell lines, and its effects were studied using cell viability assays, immunoblotting and immunofluorescence.

**Results:**

Sixty-four samples (28.7%) stained positive for Her2 (IHC 3+), and 54% (122/223) of all breast cancers stained positive for Pin1. Of the Her2-positive cancers 40 (62.5%) were also Pin1-positive, based on strong nuclear or nuclear and cytoplasmic staining. Inhibition of Pin1 via RNAi resulted in significant suppression of Her2-positive tumor cell growth in BT474, SKBR3 and AU565 cells. Pin1 inhibition greatly increased the sensitivity of Her2-positive breast cancer cells to the mTOR inhibitor Rapamycin, while it did not increase their sensitivity to Trastuzumab, suggesting that Pin1 might act on Her2 signaling. We found that Pin1 interacted with the protein complex that contains ubiquitinated erbB2 and that Pin1 inhibition accelerated erbB2 degradation, which could be prevented by treatments with the proteasome inhibitor ALLnL.

**Conclusion:**

Pin1 is a novel regulator of erbB2 that modulates the ubiquitin-mediated degradation of erbB2. The overexpression of Pin1 in a majority of Her2-overexpressing breast cancer may contribute to maintain erbB2 levels. Pin1 inhibition alone and in conjunction with mTOR inhibition suppresses the growth of Her2+ breast cancer cells.

## Background

Overexpression of the receptor tyrosine kinase HER-2/Neu occurs in up to 30% of breast cancer patients and is indicative of poor prognosis [1]. Her2/Neu plays an important causal role in breast carcinogenesis, and serves as a therapeutic target for the humanized monoclonal antibody Trastuzumab (Herceptin) [2,3]. While Her2-Neu overexpression is primarily a result of erbB2 amplification, it has recently been recognized that erbB2 levels are also regulated on the protein level [4,5]. However, factors that regulate Her2/Neu protein stability are less well understood. The prolyl isomerase Pin1 catalyzes the isomerization of specific pSer/Thr-Pro motifs that have been phosphorylated in response to mitogenic signaling. This post-phosphorylational modification can have profound effects on the stability, function and localization of the target protein [6,7] Pin1 is overexpressed in a range of human cancers [8,9], and high Pin1 expression is found in common adenocarcinomas, such as breast, lung, colon and prostate cancers [10,11]. In breast cancer, Pin1 levels are increased more in high grade than in low grade tumors [8]. A similar trend was found in prostate cancer. Ayala et al examined Pin1 levels in prostatectomy specimens from 580 prostate cancer patients and found a tight correlation of high Pin1 levels with poor prognosis [10]. Increased Pin1 levels were highly predictive of clinical failure, i.e. the development of metastatic disease in men who had undergone prostatectomy. In pre-clinical studies, Ryo et al. showed that siRNA inhibition of Pin1 inhibited both the growth of prostate cancer cell lines in vitro, and the outgrowth of prostate cancers in mouse xenotransplant experiments [12]. The association of Pin1 with an aggressive biology in both prostate and breast cancers points toward a potential tumor-promoting function of Pin1.

On the molecular level, Pin1-mediated prolyl isomerization can regulate its targets by either affecting their transcription, their stability or their function, depending on its target. Pin1 typically binds phospho-serine or phospho-threonine residues next to Proline. Upon binding with its WW-domain, Pin1 catalyzes the conversion of the adjacent prolyl residue from the cis to the trans position or vice versa. This post-phosphorylational conformational change can have profound impacts on the function, subcellular localization or stability of the target protein. Pin1 modulates several proteins that are activated downstream from erbB2, such as the AP1 complex members c-jun [8] and c-fos and cyclin D1 [13,14]. Pin1 regulates the phosphorylation status of Raf-1 kinase through regulation of the interaction with its phosphatase, PP2A. Raf-1 is responsive to receptor tyrosine kinase activation, and upon phosphorylation Raf-1 activates MEK and ERK kinases [15]. Pin1 mediated-prolyl isomerization augments various molecular functions, such as the transcriptional activity of c-fos [16]or c-jun[8,17], the localization and stability of cyclinD1[8,14,18-20] or the de-phosphorylation of Raf-1[15]. The net result of the diverse effects of Pin1-mediated prolyl isomerization of these mitotic phosphoproteins downstream from erbB2 is always accelerated progression through mitosis and cell growth.

We therefore hypothesized that inhibition of Pin1 might block the growth of Her2-positive breast cancer cells. We have previously shown that Pin1 null mice were largely protected from breast cancers induced by the c-neu transgene [13]. While 100% of the MMTV-Ras and over 90% of the MMTV-Neu transgenic mice in the wild-type Pin1 background developed one or several breast cancers within 75 weeks of observation, over 85% of transgenic mice in the Pin1 -/- background remained breast cancer-free over the same period. These *in vitro *and *in vivo *data point toward Pin1 as a potential therapeutic target in adenocarcinomas, and specifically Her2+ breast cancer [13]. However, although these studies showed that the absence of Pin1 prevented breast cancer induced by Her2 or Ras, they have not demonstrated that Pin1 inhibition can successfully treat breast cancer. Here, we report that Pin1 overexpression is found in 62% of Her2-positive breast cancer, and that Pin1 inhibition suppresses the growth of Her2-positive breast cancer cells. While Pin1-inhibition greatly increased the sensitivity of Her2-positive breast cancer cells to the mTOR inhibitor Rapamycin, it did not increase their sensitivity to Trastuzumab. Instead, Pin1-inhibition interfered with erbB2 signaling by decreasing erbB2 protein levels through an acceleration of erbB2 degradation.

## Materials and methods

### Materials

Breast cancer cell lines were purchased from the American Type Culture Collection (ATCC) and maintained in DMEM supplemented with 10% fetal bovine serum. Trastuzumab(Genentech, San Francisco) was obtained from our pharmacy. Rapamycin was from Signal Transduction Laboratories. Anonymized tissue microarrays with corresponding diagnostic information were purchased from Immunogenex. Anti-HER2 (Ab-1 and Ab-3) were obtained from Calbiochem. Actin antibodies and Cycloheximide were from Sigma. cDNA Synthesis was done using a reverse transcriptase kit from Roche (Indianapolis, IN). Vector control and DN-Pin1 (S16A) mutation under the control of the CMV promoter have been described previously [8].

### siRNA

Pin1 siRNA duplexes were purchased from and designed by Qiagen using the HiPerformance Design Algorithm licensed from Novartis. Additional siRNA duplexes directed against three other regions of the Pin1 mRNA were purchased from Invitrogen. All siRNAs led to a down-regulation of Pin1, the most effective inhibition was achieved with the target sequence CAG GCC GAG TGT ACT ACT TCA. A scrambled siRNA was used as a negative control. HiPerfect Reagent was from Quiagen and used with Opti-MEM according to manufacturer's instructions.

### Immunoblotting, RNA extraction, and immunofluorescence were done as described [13]

Immunofluorescence images were obtained using a Zeiss Confocal Laser Microscope LSM 510.

### Cell viability assay

The Promega Cell-Titer Blue Viability Assay was used to assess cell viability at the indicated time points. Cells were grown in 96-well plates and the assay performed according to the manufacturer's instructions. Absorption was read in a Perkin Elmer spectrophotometer with plate reader.

### Immunohistochemistry

Her2/neu-positive human breast cancer tissue and control slides were obtained from Zymed Laboratories (S. San Francisco, CA). Additional slides and tissue were also purchased from Tissue Array Networks. The HercepTest from DakoCytomation was used to stain for Her2, per the included protocol. Scoring was done blinded by a pathologist (BW).

## Results

### Pin1 is overexpressed in 62% of Her2-amplified breast cancer

**Figure 1:**
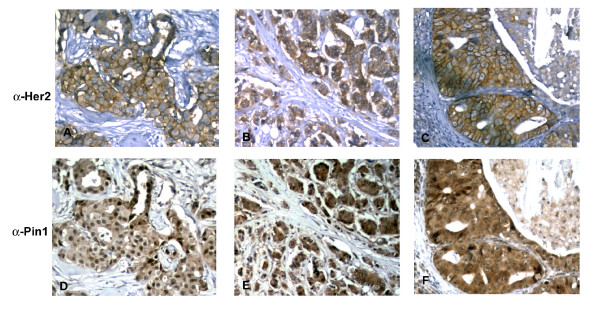
Examples of immunohistochemistry stains for Pin1 and Her2 on 3 individual tumors (in rows A through F), IHC for Her2 (A, B, C) and Pin1 (D, E, F) performed using standard procedures on consecutive slides.

As a first step, we set out to examine the frequency of Pin1 overexpression in Her2+ human breast cancer. A total of 223 breast cancer specimens, mostly from primary tumors, were analyzed by immunohistochemistry for Her2 and Pin1 expression. The intensity and distribution of the stain were graded (Tab 1). Fig. 1 provides examples of the IHC stains for Her2 (upper panels) and Pin1 (lower panels). Both stains were specific for the epithelial cancer cells with little or no no background stain in the architectural and connective tissue portions of the specimen. Cells were considered Her2-positive (3+) when a strong stain encircled the entire cell. Weak and incomplete staining were graded 1+ or 2+ and absent staining 0. Her2 1+, 2+ and 0 were considered negative. Pin1 staining was also graded according to intensity (0 – 3+). In most cancers we found nuclear and cytoplasmic localization of Pin1; however, in some cancers we saw only nuclear staining. Pin1 has been found in both nucleus and cytoplasm in cancer cells [8,10,21], and it is at this point unclear what biological significance a variation in the subcellular distribution of Pin1 may have. We found that 54% of all breast cancers were Pin1-positive (Tab. 1). 28.7% of the breast cancers were Her2-positive, which is consistent with literature reports ([22-24]), and amongst the samples that were Her2-positive in our cohort, 62.5% were also Pin1+. In summary, a majority of breast cancers overexpressed Pin1 (54%), and Pin1 overexpression was more prevalent in Her2-overexpressing tumors (62.5%) than in Her2-negative breast cancers. (Tab. 1). This observation led us to the question whether Pin1 inhibition would affect the growth of Her2+ breast cancer cells.

**Table 1:** Correlation of Pin1 and Her2 stains in breast cancer. 64/223 (28.7%) of samples were Her2-positive (3+ index). 122/223 (54%) were Pin1 + (3+ index), 40/64 (62.5%) of Her2+ breast cancer were also Pin1+.

	**Her -**	**Her2 1+**	**Her2 2+**	**Her2 3+ (pos)**	
**Pin1 -**	43	9	10	21	83

**1+**	0	1	0	1	2

**2+**	8	4	2	2	16

**3+ (pos)**	63	13	6	40	122

	114	27	18	64	223

### Pin1 inhibition suppresses the growth of Her2+ breast cancer cells

The expectation that Pin1 inhibition might suppress the growth of Her2+ breast cancer cells is based on the following pre-clinical observations: 1. Pin1 -/- mice are largely protected from breast cancers induced by the c-neu transgene [13]. 2. Pin1 is a pivotal regulator of cyclin D1 expression [7,8,13,14,25], and disruption of the cyclin D1 gene in mice also suppresses the ability of the c-Neu transgene to induce tumor development in the mammary gland [26]. 3. Pin1 inhibition has previously been shown to inhibit MAPKinase signaling [27] and signaling downstream from Raf-kinase [15].

**Figure 2:**
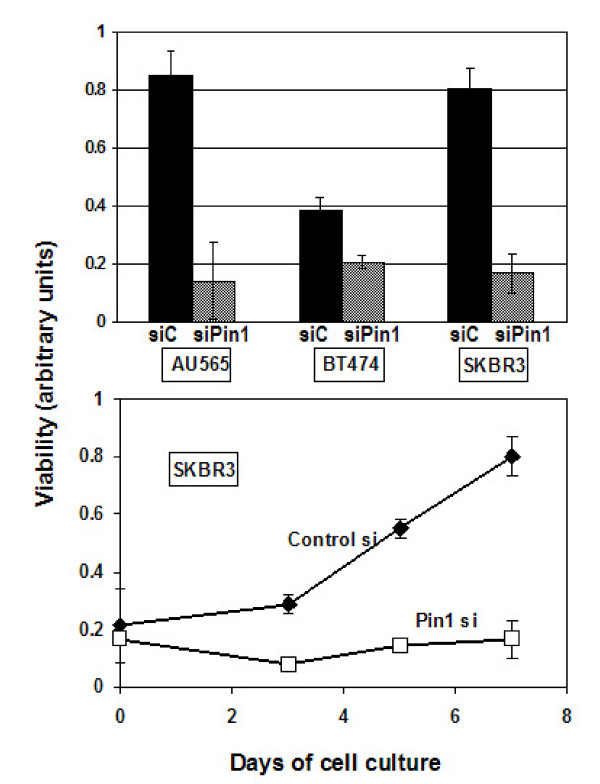
Pin1 depletion inhibits the growth of Her2+ breast cancer cell lines. Cells were transfected with control RNAi (black bars) or Pin1 siRNA (grey bars), and seeded on day 3 after transfection at 5000 cells/well in 96-well plates. Cells were allowed to grow and the resulting cultures were subjected to an MTT-based viability assay and read in a 96 well reader after 7 days (A) or at the indicated time points (B). Assays were done in triplicates.

**Figure 3:**
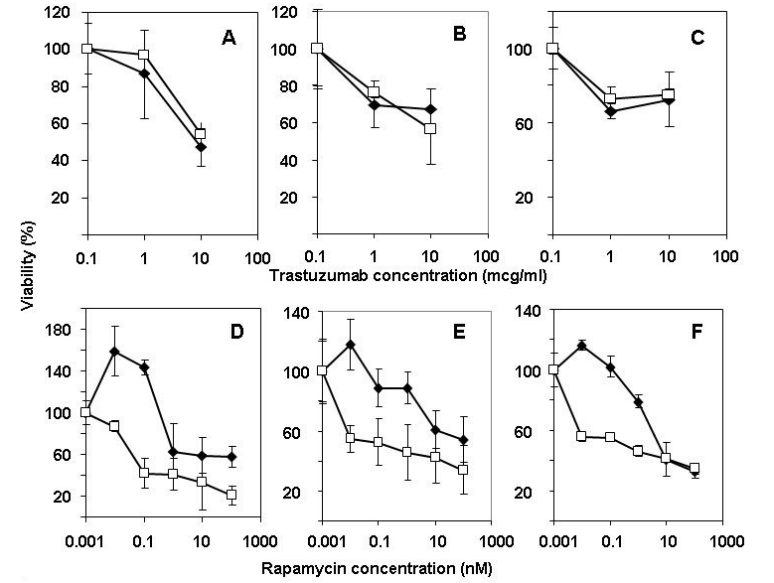
Pin1 depletion sensitizes erbB2-amplified cells to Rapamycin but not to Trastuzumab treatments. SKBR3 cells (A, D), BT474 cells (B, E) and AU565 cells (C, F) were transfected with control RNAi (dark symbols) or Pin1 siRNA (open symbols), and seeded on day 3 after transfection at 5000 cells/well in 96-well plates, and treated with the indicated concentrations of Rapamycin or Trastuzumab. Cells were allowed to grow for 5 days and the resulting cultures were subjected to an MTT-based viability assay and read in a 96 well reader. Assays were done in triplicates.

**Figure 4:**
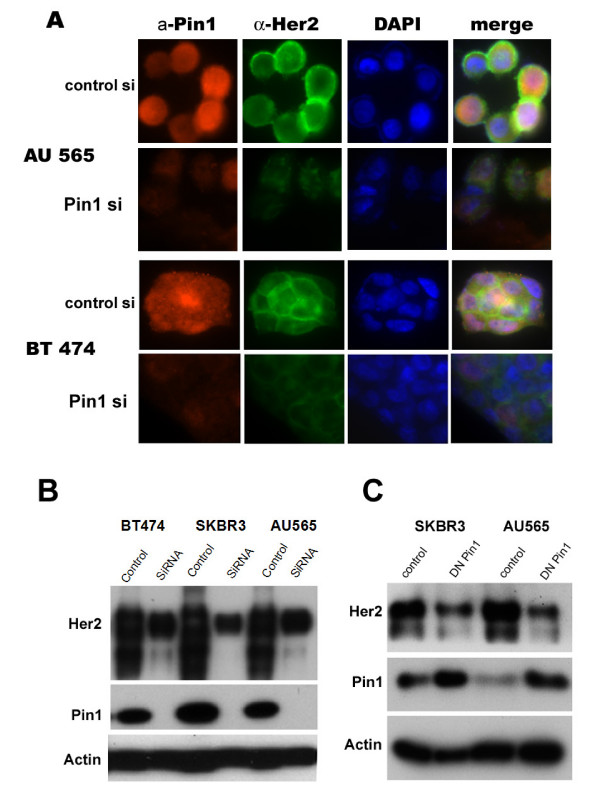
**Pin1 inhibition leads to the down-regulation of erbB2**. A. Immunofluorescence of BT474 or AU565 cells stained with anti-Pin1 (red), anti Her2 (green) and nuclear DAPI stain, and a merged image. Cells were analyzed with a Zeiss Confocal Microscope 3 days after transfection of either control or Pin1 siRNA. B, C Immunoblotting of Her2 in cells transfected with Pin1 siRNA or control siRNA (B), or dominant-negative Pin1 (S16A mutation) or control vector (C). Actin levels (lower panel) remained stable while Pin1 levels decreased in response to siRNA treatment (B, middle panel) or increased after DN Pin1 expression (C, middle panel). Her2 levels decreased both in response to Pin1 siRNA treatment (B, upper panel) and in response to expression of DN Pin1 (C, upper panel). Cells were transfected with either vector or the mutant, and lysates collected for immunoblotting 72 hrs later.

**Figure 5:**
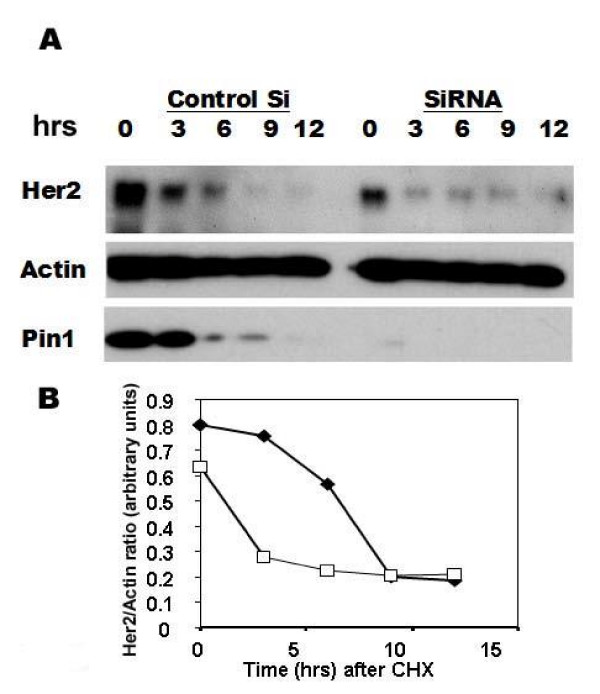
Decreased stability of Her2 after Pin1 inhibition. Her2-positive breast cancer cells (SKBR3) were treated with Pin1 control or siRNA for 3 days, and then treated with Cycloheximide at 100 mcg/ml, and lysates were obtained at the indicated time points and prepared for immunoblotting (A, B). B The intensity of the bands was quantified using ImageJ software and plotted as time versus ratio of Her2/Actin intensity for SKBR3.

To inhibit Pin1, we optimized Pin1-specific siRNA transfection with a 20-mer directed against the 5' portion of Pin1 and achieved near-complete ablation of Pin1 in all breast cancer cell lines examined within 72 hours of transfection (Fig. 2, 3, 4A, B and Fig. 5).

**Figure 6:**
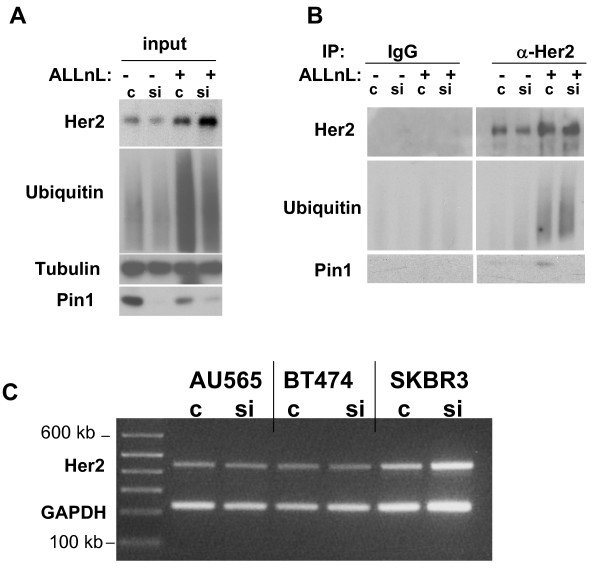
ErbB2 degradation induced by Pin1 inhibition can be rescued by proteasome inhibition. A. SKBR3 cells were transfected with control or Pin1 siRNA for 72 hours. 6 hours prior to protein lysis cells were treated with 100 μM ALLnL. B. Cell lysates were immunoprecipitated with anti-erbB2 antibodies and immunoblotting was done using antibodies against erbB2, Ubiquitin, Tubulin and Pin1. C. SKBR3, AU565 and BT474 were transfected with control or Pin1 siRNA for 72 hours. RNA was extracted using Trizol reagent and RT-PCR was performed using erbB2 and GAPDH specific primers.

To examine the effect of Pin1 inhibition on the growth pattern of breast cancer with Her2-amplification, we used siRNA inhibition of Pin1 in the Her2-positive cell lines AU565, BT474 and SKBr3. We plated these cells on day 3 after transfection at equal densities. After 4 more days, viability was assessed using the MTT assay, continued Pin1 inhibition was confirmed with immunoblotting. Consistent with prior reports, Pin1 inhibition slowed down cell growth considerably (Fig. 2). Within 4 days in culture, control-transfected cells grew exponentially, while the Pin1 si-RNA treated cells grew very slowly (Fig. 2). In summary, Pin1-inhibition led to a consistent inhibition of cell growth in all three Her2-positive cell lines examined.

### Pin1 inhibition enhances the growth-inhibitory effects of Rapamycin but not of Trastuzumab

Resistance to erbB2-directed cancer treatments is an unsolved clinical problem [28,29], and therefore we asked if Pin1 inhibition could enhance the efficacy of other signaling inhibitors that target erbB2-dependent pathways. The monoclonal antibody Trastuzumab is FDA-approved for the treatment of breast cancer and inhibits the homodimerization of ErbB2 [28,30]. Rapamycin and its analogs, which are currently in late-stage clinical trials in breast cancer, inhibit mTOR kinase, a key regulator of cyclin D1 [31,32]. Both Trastuzumab and Rapamycin are clearly effective inhibitors of ErbB2-mediated signaling; however, they do not block breast cancer cell growth *in vitro *[29,33] or *in vivo *[30] completely but rather attenuate cancer cell growth. We therefore asked if Pin1 inhibition could augment the efficacy of these drugs *in vitro*. We transfected AU565, BT474 and SKBr3 cells with control or Pin1 siRNA, and after 3 days we seeded them out at equal densities and treated these cells with Trastuzumab and Rapamycin. After 4–7 more days, cell viability was assessed using the MTT assay, and the data were normalized to controls (vehicle-treated only). We found that the Her2+ cell lines AU565, BT474 and SKBR3 were modestly responsive to therapeutic doses of Trastuzumab *in vitro *at a range of 1–10 mcg/ml that correspond to therapeutic trough levels [34]. Unexpectedly, Pin1-inhibition did not increase the sensitivity of any of the Her2+ cell lines to Trastuzumab (Fig. 3A, B, C). Recently, concurrent Her2-directed and mTOR-directed treatments have been proposed to overcome Trastuzumab sensitivity [35]. As reported earlier [36], the Her2+ cell lines were also sensitive to Rapamycin in the range of 1–10 nM. In contrast to Trastuzumab sensitivity, Rapamycin sensitivity increased substantially after Pin1 inhibition (Fig. 3D, E, F), rendering the cells sensitive to doses as low as 0.1 nM of the mTOR inhibitor. In summary, we found that Pin1-inhibition sensitized Her2-positive breast cancer cells to the mTOR-inhibitor Rapamycin, but not to the direct erbB2 inhibitor Trastuzumab.

### Pin1 inhibition decreases erbB2 levels

We then asked if Pin1 affected components of the erbB2 signaling cascade other than cyclinD1, starting with erbB2 itself. Surprisingly, we found that Pin1-inhibition led to a decrease in erbB2 levels in all three Her2-positive cell lines starting 72 hours after Pin1 inhibition (Fig. 4 and 5). Immunoblotting showed near-complete ablation of Pin1 after 72 hours (Fig. 4A, B, Fig. 5), with concomitant decrease in erbB2 levels, while Actin levels stayed unaffected. The effect of Pin1-inhibition on Her2 levels was not only seen in immunoblots but also on the single-cell level using immunofluorescence (Fig. 4A). In AU565 and BT474 cells, the Pin1 stain is a diffuse nuclear and cytoplasmic stain (red) while the Her2 stain encircles the cytoplasmic membrane (green). After Pin1 inhibition (lower panel), the Her2 stain is only a scant membrane and cytoplasmic stain (Fig. 4A). DAPI stain was used to visualize the nuclei. To ensure that our findings were not an off-target effect of the siRNA, we used alternate Pin1 siRNAs as well as over-expression of a dominant-negative mutant of Pin1 (Pin1 S16A, [13]), and, using this alternate mechanism to suppress Pin1's function, we saw again a decrease in erbB2 levels when the Pin1S16A mutant was overexpressed (Fig. 4C).

### Pin1 inhibition leads to decreased Her2 levels through a post-transcriptional mechanism

Her2's function is thought to be regulated primarily through tyrosine phosphorylation, while Her2 protein levels are regulated by transcriptional mechanisms [37] as well as by proteasome-mediated degradation [38,39]. Pin1-mediated prolyl isomerization regulates proteasomal degradation of some of its target proteins, such as the degradation of c-myc[40] and Cyclin E[41], and prevention of degradation of cyclin D1[14,25]. However, a regulatory role of Pin1 for a receptor tyrosine kinase such as erbB2 has thus far not been reported. Therefore, we examined if Pin1 inhibition affected the stability of erbB2. In cells treated with control and with Pin1 siRNA inhibition, we followed erbB2 levels after inhibition of protein biosynthesis with Cycloheximide (Fig. 5A, B). As reported earlier [38], erbB2 levels in breast cancer cells treated with Cycloheximide started to decrease within 3 to 6 hours of Cycloheximide treatment, and continued to drop after 9 hours [38]. ErbB2 levels before Cycloheximide treatment were already lowered because of Pin1 siRNA treatment in SKBR3 (Fig. 5A) and AU565 and BT474 (not shown). However, in Pin1 siRNA-treated SKBR3 cells where erbB2 was still detectable after Pin1 siRNA treatment, (Fig. 5A, B), erbB2 levels decreased precipitously within 3 hours after Cycloheximide treatment, indicating an accelerated degradation of erbB2 in the absence of Pin1.

To determine how Pin1 depletion accelerated erbB2 degradation, we examined the ubiquitination of erbB2. We used the proteasome inhibitor ALLnL to stabilize ubiquitinated erbB2 for 6 hours prior to lysis of the cells. As expected, the levels of erbB2 and of ubiquitination increased substantially after treatment with the proteasome inhibitor (Fig. 6A). In co-immunoprecipitation with erbB2 antibodies we found that the increase in erbB2 is indeed accompanied by an increase in erbB2 ubiquitination (Fig. 6B). Interestingly, we found that ALLnL treatment prevented the ubiquitin-mediated degradation of erbB2 induced by Pin1 depletion (Fig. 6), and that Pin1 itself co-precipitated with the protein complexes that contain ubiquitinated erbB2 (Fig. 6B). Finally, we asked if Pin1 inhibition affected erbB2 mRNA levels (Fig. 6C). We used a semi-quantitative RT-PCR approach, and found that erbB2 transcription did not differ in control and Pin1 siRNA-treated cells. In summary, our data suggest that Pin1 regulates erbB2 stability by interfering with its proteasomal degradation.

## Discussion

Her2-positive breast cancer presents a special clinical problem as the amplification of Her2 on the one hand is a clear indicator of poor prognosis, while on the other hand it is also a clear predictor of response to Her2-directed therapies [42]. The humanized monoclonal antibody trastuzumab (Herceptin) is initially clearly effective as mono- and in combination treatments. However, almost all patients develop resistance and the disease eventually progresses on trastuzumab [42], making the development of strategies to overcome Trastuzumab resistance urgent. To evaluate Pin1 as a potential treatment target for Her2+ breast cancer, we set out first to examine the prevalence of Pin1 expression in Her2-positive breast cancer, and found co-expression in 62% of Her2+ breast cancer specimen, suggesting that simultaneous inhibition of Pin1 and Her2 might be effective (Fig. 1, Tab 1). Consistently, all three Her2+ cell lines that we examined, were strongly inhibited in their growth by Pin1 inhibition (Fig. 2).

We had previously shown that Pin1 -/- mice are largely protected from the tumorigenic effects of oncogenic c-Neu or v-Ha-Ras, but not c-Myc [13], as is the case for cyclin D1 null mice [26], and that Pin1 ablation is effective in preventing oncogenic Neu or Ras from inducing cyclin D1 in mice [13]. These data confirmed the dependence of Her2-induced tumor growth on cyclin D1 phosphorylation [43] and the decisive modulatory role that Pin1 plays in the regulation of this signaling cascade [8,13,14,25]. It is important to note, however, that in the mouse model, we showed that Pin1 ablation prevented breast cancer [13], while in the current experiments we attempted to inhibit the growth of actual cancer cells. We used Pin1 siRNA inhibition to down-regulate Pin1 in Her2-positive breast cancer cells. The down-regulation was achieved within 72 hours, and lasted at least for seven days. Because of these kinetics, the siRNA inhibition likely mimics a pharmacological inhibition of Pin1 better than the genetic absence of Pin1, and our results indicate that siRNA inhibition of Pin1 is highly effective in inhibiting tumor cell growth of Her2+ breast cancer cells (Fig. 2).

Recent work on the mechanism of trastuzumab-mediated growth arrest and on trastuzumab resistance has shown that in tumors that are trastuzumab-resistant, direct inhibition of one or several of downstream targets of Her2 may help to overcome resistance [44]. The serine/threonine kinase mTOR (mammalian Target of Rapamycin) is a key integrator of multiple cell stimuli, such as growth factor and cytokine signals, but also nutrient and energy status. Furthermore, signals emanating from mTOR regulate cell growth, proliferation and survival [31,45]. Inhibition of the mTOR pathway is effective in inhibiting tumor growth in cell lines [46] (Fig. 3) and in xenograft models of erbB2-overexpressing breast cancer [47] and is currently under investigation in clinical trials. Consistently, both, Trastuzumab and the mTOR-inhibitor Rapamycin, inhibited the growth of Her+breast cancer cell lines. Interestingly, we found that simultaneous Pin1 inhibition increased the sensitivity of these cells to Rapamycin (Fig. 3). The sensitization of Her+ breast cancer cells to Rapamycin by Pin-inhibition was expected as Pin1 regulates signaling both up- and downstream from mTOR, thereby likely rendering these cells more vulnerable to growth arrest as a consequence of Pin1 inhibition. On the other hand, Pin1 inhibition did not increase the sensitivity of these cells to Trastuzumab. This may be explained by our finding that Pin1 induces the degradation of erbB2, thereby reducing the levels of the Trastuzumab binding sites (Fig. 4, 5).

Our data indicate that Pin1 is a binding partner of the erbB2 protein complex, and that erbB2 degradation induced by Pin1 inhibition can be rescued by proteasome inhibition (Fig. 6). There is precedent for the interaction of Pin1 with proteins that subsequently undergo ubiquitin-mediated degradation: The interaction of Pin1 with p53 protects phosphorylated p53 from interaction with its ubiquitin ligase, MDM2 [48,49] and Pin1's interaction with the transcriptional activator Che1 [50], which promotes interaction with HDM2 and subsequent degradation of Che1. Pin1's exact binding site in the erbB2 protein complex is yet to be determined. The amount of Pin1 that co-immunoprecipitated increased substantially when we prevented proteasome-mediated degradation of erbB2 that led to the accumulation of ubiquitinated erbB2. These findings strongly support a role of Pin1 for the protein stabilization of erbB2. Threonine or Serine phosphorylation has only recently been found to occur in erbB2 in response to phorbol-ester or growth factor treatment [51,52]. The effects of threonine or serine phosphorylation on the function or stability of ErbB2 are largely unknown. Two putative phosphorylation sites are flanked by a proline, and they are therefore potential Pin1 binding sites, Threonine 701 and Serine 1174. We are currently investigating if Pin1-mediated prolyl-isomerization at T701 and/or S1174 could affect the ubiquitination of erbB2, its kinase activity or its ability to assemble with the triage complex erbB2-CHIP-HSP90-HSP70 [39]. It is also possible that Pin1 affects erbB2 stability not through direct interaction but indirectly through modulation of the respective Ubiquitin-ligase or some other modification of the proteasome complex.

The development of Prolyl-isomerase inhibitors as cancer therapeutics is still at an early stage. The only inhibitors of prolyl isomerization currently in clinical trials are the mTOR inhibitors. These Rapamycin analogs are direct inhibitors of the prolyl isomerase FKBP12 [31,53], which then inhibits mTOR, and are in clinical trials in kidney, breast and lung cancer [47,54]. Pin1 itself has been proposed as a potential therapeutic target, but aside from Juglone [55,56], specific inhibitors have to date not yet been identified. Knowing which tumors express Pin1 highly and therefore are "Pin1-inhibitor-sensitive" may help assess, which tumor types might respond to Pin1-directed treatments. For therapeutic purposes, the diversity of signaling cascades that Pin1 is involved in may be an advantage and provide the broad inhibitory coverage of targets that may be needed to treat cancer efficiently [57]. Our data suggest that inhibition of phospho-specific prolylisomerization by Pin1 may provide a way of simultaneously blocking multiple signal transduction pathways and enhancing the efficacy of specific target-directed medications.

## Competing interests

The authors declare that they have no competing interests.

## Authors' contributions

PBL carried out most of the experiments including the immunohistochemistry and immunofluorescence stains, cell viability assays and immunoblotting in response to treatments and drafted the manuscript. LB and EH participated in cell culture, RT-PCR and immunoblotting experiments. BW read IHC slides. KPL participated in the design of the study and provided critical reagents. GMW conceived of the study, participated in its design and coordination, participated in the experiments and edited the manuscript. The authors read and approved the final manuscript.
